# Functional magnetic particle imaging (fMPI) of cerebrovascular changes in the rat brain during hypercapnia

**DOI:** 10.1088/1361-6560/acecd1

**Published:** 2023-08-28

**Authors:** Erica E Mason, Eli Mattingly, Konstantin Herb, Stephen F Cauley, Monika Śliwiak, John M Drago, Matthias Graeser, Emiri T Mandeville, Joseph B Mandeville, Lawrence L Wald

**Affiliations:** 1 A. A. Martinos Center for Biomedical Imaging, Dept. of Radiology, Massachusetts General Hospital, Charlestown, MA, United States of America; 2 Harvard-MIT Division of Health Sciences & Technology, Cambridge, MA, United States of America; 3 Harvard Medical School, Boston, MA, United States of America; 4 ETH Zurich, Department of Physics, Zurich, Switzerland; 5 Massachusetts Institute of Technology, Department of Electrical Engineering & Computer Science, Cambridge, MA, United States of America; 6 Fraunhofer Research Institution for Individualized and Cell-Based Medical Engineering, IMTE, Lübeck, Germany

**Keywords:** Magnetic particle imaging (MPI), functional magnetic particle imaging (fMPI), superparamagnetic iron oxide nanoparticles (SPION), cerebral blood volume (CBV), hypercapnia, contrast-to-noise ratio (CNR)

## Abstract

*Objective.* Non-invasive functional brain imaging modalities are limited in number, each with its own complex trade-offs between sensitivity, spatial and temporal resolution, and the directness with which the measured signals reflect neuronal activation. Magnetic particle imaging (MPI) directly maps the cerebral blood volume (CBV), and its high sensitivity derives from the nonlinear magnetization of the superparamagnetic iron oxide nanoparticle (SPION) tracer confined to the blood pool. Our work evaluates functional MPI (fMPI) as a new hemodynamic functional imaging modality by mapping the CBV response in a rodent model where CBV is modulated by hypercapnic breathing manipulation. *Approach.* The rodent fMPI time-series data were acquired with a mechanically rotating field-free line MPI scanner capable of 5 s temporal resolution and 3 mm spatial resolution. The rat’s CBV was modulated for 30 min with alternating 5 min hyper-/hypocapnic states, and processed using conventional fMRI tools. We compare our results to fMRI responses undergoing similar hypercapnia protocols found in the literature, and reinforce this comparison in a study of one rat with 9.4T BOLD fMRI using the identical protocol. *Main results.* The initial image in the time-series showed mean resting brain voxel SNR values, averaged across rats, of 99.9 following the first 10 mg kg^−1^ SPION injection and 134 following the second. The time-series fit a conventional General Linear Model with a 15%–40% CBV change and a peak pixel CNR between 12 and 29, 2–6× higher than found in fMRI. *Significance.* This work introduces a functional modality with high sensitivity, although currently limited spatial and temporal resolution. With future clinical-scale development, a large increase in sensitivity could supplement other modalities and help transition functional brain imaging from a neuroscience tool focusing on population averages to a clinically relevant modality capable of detecting differences in individual patients.

## 1. Introduction

Magnetic particle imaging (MPI) (Gleich and Weizenecker 2005) is a rapidly developing preclinical imaging modality with emerging efforts toward human scanners (Mason *et al* 2017, Graeser *et al* 2019, Vogel *et al* 2022) that directly images an injected superparamagnetic iron oxide nanoparticle (SPION) tracer. MPI has been proposed as a functional neuroimaging modality for imaging the cerebral blood volume (CBV) modulations associated with brain activity (Mason *et al* 2017, Cooley *et al* 2018, Herb *et al* 2020). MPI has positive contrast with no biological background signal (there are no endogenous signal sources present in the body), high sensitivity (due to the high magnetic moment of the tracer), and fast imaging times. MPI lacks, compared to MRI, the ability to acquire multiple types of anatomical contrast, flexibility in trading sensitivity and spatial resolution, and the thousands of person-years of engineering found in today’s widely available commercial MRI scanners.

While MRI is a versatile tool with many useful features, including the ability to image with or without tracers, its indirect contrast mechanisms also impose limitations. Because fMRI detects functional changes as an indirect and relatively small modulation (≈1%) of the already weak nuclear magnetic resonance signal from the water protons, it is fundamentally limited in sensitivity. This originates from the size of the proton nuclear magnetic moment and contamination from unwanted physiological modulations of the background signal. The former effect is addressed in MPI by detecting the much higher moment magnetic of the SPION tracer (compared to the proton’s magnetic moment), and background signal modulations are reduced in MPI by imaging a tracer confined to the compartment of interest: the CBV.

With the tracer confined to the blood pool, MPI directly probes the blood content of a voxel, without background from other tissue compartments. The signal changes then reflect the full modulation of CBV by brain activation (a ∼25% effect for robust brain activation) and the MPI signal is expected to increase proportionally to CBV. The lack of background signal from the extravascular compartments (which constitute 95% of the fMRI signal) suggests that a blood tracer method will also incur significantly less physiological noise, which can be thought of as a nuisance modulation of signal from this irrelevant compartment and is often the dominant noise source in fMRI (Krüger and Glover 2001). Furthermore, the physiological noise retained from the blood pool compartment is potentially directly relevant to functional connectivity mapping through ‘noise’ correlations between functionally connected brain regions (Biswal *et al* 1995, Fox *et al* 2005).

In this work, we report the first use of time-series MPI for imaging hemodynamic modulation in the brain (termed functional MPI, ‘fMPI’) that probes the modality’s sensitivity and limitations. While fMPI will likely never be a replacement for preclinical rodent fMRI due to its comparative limitations in spatial resolution, demonstration of the first rodent fMPI time-series imaging is an important stepping stone to assess fMPI as a potential clinical tool. We present 2D time-series *in vivo* MPI imaging of rats undergoing a hypercapnia activation paradigm, expanding beyond our proof-of-concept pilot imaging trial (Herb *et al* 2020) with critically improved MPI hardware for increased sensitivity, thermal stability, temporal resolution, and image reconstruction. We analyze the experimental contrast-to-noise ratio (CNR) of the hypercapnia challenge in five rats and compare to fMRI results for similar hypercapnia challenges in the literature. Additionally we show 9.4T fMRI rat data for a single animal using the identical CBV modulation protocol. The experiment serves as a demonstration of our specific hypercapnia protocol in a standard preclinical fMRI sequence. It does not account for all fMRI variants (different field strengths, CBV fMRI, etc.) and is insufficiently statistically powered to make broad conclusions (only a single animal). We therefore rely on the plentiful data available in the preclinical fMRI literature for our comparison of fMPI and fMRI CNR.

### 1.1. Magnetic particle imaging principles

MPI detects and spatially localizes the nonlinear magnetization response of an injected SPION tracer to an applied AC magnetic field (the ‘drive’ field) (Gleich and Weizenecker 2005). The temporal response of the SPION magnetic moment includes a nonlinear component comprising harmonic terms not present in the applied drive field. The SPION’s modulated magnetic moment is detected by one or more receiver coils via Faraday detection. The direct inductive pickup from the drive field itself is filtered out leaving only the SPION-induced harmonic signals which are digitized to form an image. In the presence of an applied static gradient field, the SPION magnetic moment is saturated at all locations outside of a field-free region (near the zero crossing of the gradient field), eliminating signal detection from these regions. The field-free region is either a field-free point (FFP) (Gleich and Weizenecker 2005) or field-free line (FFL) (Weizenecker *et al* 2008), and it can be moved rapidly in space with the application of an additional, uniform field from ‘shift’ or ‘focus’ coils. This sensitive region is scanned around the field of view (FOV) to acquire an image, which is reconstructed using either a forward model inversion of the recorded harmonic data (system matrix MPI (Rahmer *et al* 2012)) or mapped to the FFP’s expected location (x-space MPI (Goodwill and Conolly 2011)). In the case of an FFL imager, the signal derives from the integral of the tracer concentration along the line. Sweeping the FFL across the object then forms a 1D projection image, and a 2D projection reconstructed image is formed after repeated rotated projections. In this work, we used a mechanically rotating FFL imager (Mattingly *et al* 2022).

Like MRI, MPI detects oscillating magnetic moments (d*M*/d*t*) in the body through Faraday’s law of induction. Unlike MRI, for which the magnetization derives from precessing proton nuclear magnetic moments, the magnetic moment detected in MPI is that of an injected SPION. The magnetic moment of a single SPION nanoparticle (calculated assuming a 25 nm spherical particle of Fe_3_O_4_ with magnetic moment per mass of 110 Am^2^ kg^−1^ (that of Synomag-D (Micromod Partikeltechnologie GmbH (Micromod 2022))) is ∼10^8^ times greater than that of the proton. This facilitates high sensitivity mapping of the SPION tracer. The magnetic moment strength advantage is partially offset by lower Fe concentration (140 *μ*M) compared to brain water (50 M), and additionally by the lower oscillation frequency of the moment (127 MHz for 3T MRI while MPI typically detects harmonic signals only up to approximately 1 MHz). A further difference between the two modalities is that biological tissue is magnetically linear (paramagnetic or diamagnetic) and does not produce MPI signal. Since injected SPION remains in the brain’s vasculature, the background signal from non-blood compartments is eliminated. This focus on the component of interest (the blood pool) is useful for hemodynamic-based functional imaging since it reduces background intensity drifts and modulations that could otherwise confound the measurement of small hemodynamic signal changes.

The SPION’s magnetization response to an applied field is characterized by the Langevin curve and contributes to many important aspects of MPI including the imaging resolution and sensitivity. For high MPI resolution, a SPION with a sharp transition in its magnetization curve is desired so that the signal is localized to a small region around the zero crossing of the FFL or FFP. For high sensitivity, a large saturation magnetization is needed since the detected d*M*/dt derives from the driven oscillation between the positive and negative saturated states. Thus, development of improved MPI tracers is an ongoing effort (Vreeland *et al* 2015, Lu *et al* 2021, Tay *et al* 2021). Our work used a multi-core nanoflower dextran-based SPION agent (Synomag-D 70 nm SPIONs, Micromod, Germany, item #104-124-701) with a PEG 25.000-OMe coating extending its diameter to 70 nm to prolong the blood half-life. SPIONs can be cleared fairly rapidly by the liver; in rodents this can occur with a blood half-life as short as seconds (Keselman *et al* 2017). We measured an average rat blood half-life of 48 min for our dextran/PEG coated agent.

### 1.2. CBV-based functional imaging

The potential to use cerebral hemodynamic measurements to elucidate the workings of the brain was suggested in the late 19th century by Roy and Sherrington as well as William James, who reported Angelo Mosso’s experiments measuring CBV alterations during cognitive tasks (Sandrone *et al* 2014). The first functional brain mapping performed with MRI used a non-endogenous intravascular magnetic tracer (gadolinium) to alter the MR dephasing times (*T*2*) around vessels, indirectly reporting the CBV changes accompanying human brain activation (Belliveau *et al* 1991). Non-bolus, long blood half-life dextran-coated SPION contrast agents were later applied for CBV-based rodent fMRI (Mandeville and Marota 1999, Mandeville 2012) and alert non-human primate studies (Leite *et al* 2002). More recently, fMRI based on iron oxide nanoparticles has been explored for human fMRI studies using ferumoxytol (Christen *et al* 2013, Qiu *et al* 2012). All of these fMRI methods used the injected agent as an indirect reporter of CBV by the blood-pool agent’s effect on local *T*2* relaxation. MRI methods using endogenous forms of CBV contrast and non-*T*2* mechanisms have also been introduced to facilitate high resolution fMRI in high magnetic field scanners (Huber *et al* 2017).

MPI is a natural modality for imaging CBV since SPIONs are directly detected by MPI and they remain confined to the blood pool in a healthy mammalian brain. Initial work has focused on imaging rodent cerebral vasculature (Lu *et al* 2013) as well as basal flow and CBV in rodents using a bolus injection (Ludewig *et al* 2017, Orendorff *et al* 2017, Zheng *et al* 2017). MPI’s expected sensitivity to functional CBV changes has been modeled in rodents and humans (Mason *et al* 2017) and detected in rodents in a non-imaging experiment of CBV changes following hypercapnia (Cooley *et al* 2018).

**Figure 1.**
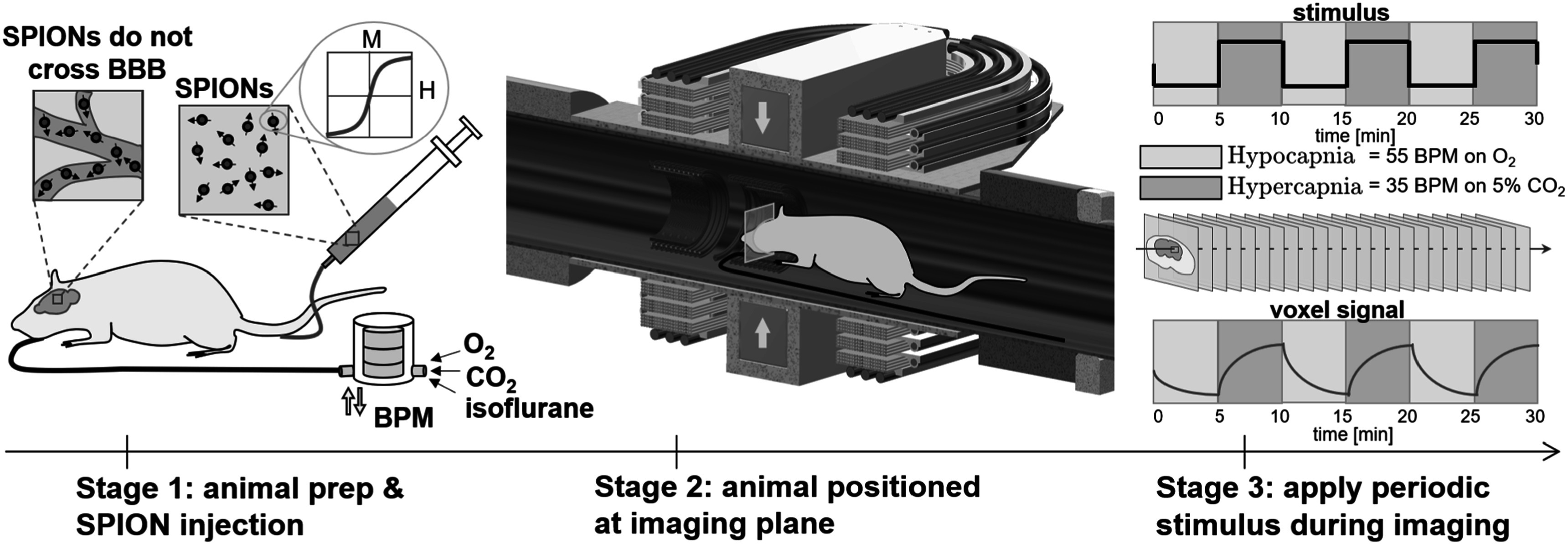
Overview of the experimental protocol. Stage 1 consists of animal preparation, including placement of arterial and venous lines, a tracheotomy, intubation and application of isoflurane anesthesia. This is followed by an intravenous injection of SPION tracer, whose nonlinear magnetic signature is imaged with MPI. Stage 2 is positioning the rat in the MPI scanner. The rat is placed on a custom bed and moved into the imager, positioning the 2D imaging slice. Stage 3 is acquisition of MPI images every 5 s, during which the gas mixture supplied to the ventilation system is modulated to induce a ΔCBV effect in the brain over time. The breathing stimulus is alternating periods (*T*/2 = 5 min) of hyper- and hypocapnia by modulating breathing rate and the %CO_2_ inhaled.

In this work, we validate the potential of fMPI to sensitively map CBV modulations by imaging rats undergoing alternating states of hyper- and hypocapnia to modulate blood volume in a paradigm similar to earlier fMRI validations (Mandeville *et al* 1998). Inhalation of gas with modulated CO_2_ or O_2_ levels was applied to elicit intervals of hyper- and hypocapnia and induce global brain CBV modulation of up to ∼25% for gas inhalation protocols used in the literature (Mandeville *et al* 1998, Wu *et al* 2002). Figure 1 shows an overview of the fMPI imaging experiment. Briefly, after application of anesthesia, tracheostomy and connection of the ventilation apparatus, rodents were injected with the long blood half-life SPION agent and imaged using MPI during the alternating 5 min cycles of hyper- and hypocapnia. The images were analyzed with a general linear model (GLM) to determine the hemodynamic changes.

## 2. Methods

### 2.1. MPI scanner and image acquisition

Magnetic particle imaging was performed using a previously described (Mattingly *et al* 2022) home-built rodent FFL MPI scanner, the design of which has been made available via the Open-Source MPI project (Mattingly *et al* 2020). It was designed for sensitive, stable, continuous rotation allowing time-series imaging over multi-hour experiments. The system employs a permanent magnet-based, mechanically rotating field-free line (FFL), a solenoid drive coil for application of the 25 kHz drive field and solenoid gradiometer receive coil with 5.4 cm opening for the rodent head. Projections at each of 27 rotation angles are taken to form the 2D image. Continuous rotation of the gantry for time-series imaging is achieved by powering and cooling the shift coils through electrical slip rings and a rotary water union. The 0.1 Hz rotation of the system provides a 2D image every 5 s continuously for as long as 2 hours. Currently the reconstruction produces a single 2D ‘slice’ per bed position, but 3D scans could be acquired with longer scan times.

The drive coil produces an 8 mT_pk_ field in the *z* direction at 25 kHz. The FFL has a gradient *G_z_* = 2.83 T m^−1^, and the shift coils sweep this with a triangular current of 42 A_pk_ at 2.7 Hz, forming a projection every 184.8 ms at an in-plane field of view of 30 mm (diameter). Note that for rat #5, we expanded the FOV to 33.6 mm to accommodate its head-position which sat high toward the edge of the FOV. Each projection is sampled at 66 points in space providing a discretization of 0.45 mm. Native spatial resolution is determined by the strength of the scanner’s field-free line (2.83 T m^−1^) and the SPION’s magnetization curve properties; the native point spread function was measured to have a FWHM of 3 mm in-plane (Mattingly *et al* 2022). The images are therefore later smoothed to the native resolution of 3 mm.

Special attention was placed on the time-series stability of the system. The drive filter was optimized to minimize its temperature coefficient, providing a drive current stability (magnitude and phase) of within 1% over 30 min with no active feedback. Continuous 5 s images were acquired in sets of 5 images, with a half rotation (5 s) gap between sets, such that each set can re-trigger on the gantry home switch for improved angular accuracy. This yields an 83% duty cycle. The gantry rotation was tracked using an optical ‘home’ switch as well as a rotation encoder, which was reset at the start of each set by the home switch.

The duty cycle of the system is high (83%) requiring attention to thermal control of the drive filter, drive coil and shift coils. The air-cooled filter inductors rose no more than 15 °C and the water-cooled drive coil was manually controlled to within 10 °C above the coolant water temperature. For the animal experiments, the water temperature was maintained between 15 °C and 20 °C to keep the bore temperature within 25 °C–30 °C.

### 2.2. Animal experimental procedure

We studied six adult male Spraque Dawley rats (5 using fMPI and 1 using fMRI), #1–6 weighing [216, 297, 338, 324, 250, 250] g. The animal preparation protocol followed our previous work (Mandeville *et al* 1998, Cooley *et al* 2018, Herb *et al* 2020). These experiments were carried out under an approved protocol from our Institution’s animal ethics committee and adhered to ARRIVE guidelines. The sex of the animals was not considered as a variable in this technological exploration, and literature shows minimal or no sex dependence in hypercapnia response (Gargaglioni *et al* 2019). During surgical preparation, anesthesia was induced with 3% isoflurane. The femoral venous and arterial catheters were inserted, and a tracheotomy was performed to enable ventilation control. The animals were placed on the removable MPI scanner bed platform, where they remained for the duration of the experiment (including transfer to the MRI scanner). Their heads were fixed using a bite-bar and tape. During the fMPI experiment, anesthesia was maintained by 1%–1.5% isoflurane and paralysis maintained with a continuous infusion of 2 ml kg^−1^ hr^−1^ pancuronium in 5 ml kg^−1^ hr^−1^ saline.

After preparation of the animal on the MPI bed, it was moved into the imaging bore such that the imaging plane was positioned to approximately the bregma. A dose of 10 mg Fe per kg dextran/PEG-coated Synomag-D 70 nm SPIONs (Micromod, Germany) was injected at a rate of 14 ml hr^−1^. Undiluted Synomag-D contains 6 mg Fe per ml of solution. Availability of the particles necessitated the use of 2 batches of SPIONs (Lot #s: 16321104-02 for rat #1, and 08522104-01 for rats #2–5). The injection is followed by a saline flush to clear the catheter dead-space and administer the full dose to the animal. The animal then underwent 3 cycles of alternating hyper-/hypocapnia modulation, for a total of 30 min. Hypercapnia was induced by ventilating the rat with 5% CO_2_ + enriched (30% O_2_) air at 35 breaths per minute (BPM); hypocapnia was induced by hyperventilation (55 BPM) on the enriched air. Each state lasted 5 min (cycle period = 10 min), and the 30 min experiment recorded the MPI signal over three full cycles (30 min total). This was followed by a second injection and another three-cycle (30 min) experiment. A switch time-stamped in synchronization with the imaging sequence was toggled to record the current activation state (hyper/hypo) throughout the experiment. Each rat also underwent a volume MPI scan, by translating the animal bed along the bore in ∼0.6 mm steps. No animals that underwent this experimental protocol were excluded from the analysis or from the presented results.

### 2.3. MRI overlay and functional images

After completion of the fMPI experiment, the animal was sacrificed and carried on the removable MPI bed for imaging in the 3T MRI scanner. The MRI data was obtained for each rat on the same bed used in the MPI system (3D GRE, TE = 2.67 ms, TR = 20 ms, isotropic 0.5 mm). The MR images were then coregistered to the MPI using the MRI visible fiducials.

One Sprague-Dawley rat was imaged with a 3D BOLD sequence (TE = 15 ms, TR = 2.5 s, 0.375 mm × 0.375 mm × 0.75 mm resolution). Hyper- and hypocapnia were induced as previously described, alternating every 5 min for 30 min. The same GLM was used in the functional time-series analysis, however with the SPION decay term (${e}^{t/{\tau }_{2}}$) removed as the BOLD effect does not rely on an injected contrast agent. The data is smoothed to 1 mm as is commonly done in preclinical fMRI, and to 3 mm for comparison to the fMPI data.

### 2.4. Image reconstruction

The imager acquires 1D projections at different angles by rotating the FFL in the *x*–*y* plane, enabling reconstruction of a 2D image. Details of the projection formation are described further in Mattingly *et al* (2022). This acquisition has a point spread function (PSF) in the through-plane dimension (*z*) due to the drive field’s orientation along this direction, as described in Mason *et al* (2022). This PSF extends about 7 mm in *z* and has regions with opposing polarity which vary spatially for each harmonic; these out-of-plane regions are referred to as ‘side-lobes.’ If signal from SPIONs at the side-lobes are allowed to contribute to the integrated signal but are assumed to originate within the 2D plane, they can constructively and destructively interfere with in-plane signal. For fast (5 s) time-series imaging with our current scanner, it is not feasible to acquire full 3D volumes at each time point. We instead developed a forward model reconstruction (Mason *et al* 2022) that suppresses signal interference from out-of-plane SPIONs which would otherwise contribute to the side-lobes of the through-plane spatial PSF. This method utilizes a 3D model of the system with projection data at multiple harmonics to encode the compressed data in the third dimension. While not used to reconstruct a full 3D image, it instead informs the removal of out-of-plane interference artifacts in the center 2D image.

The forward model of the measured MPI projection data is formed from a hybrid measured/simulated model of the system. It is specific to the hardware’s applied fields and measured SPION magnetization curve. The resulting linear systems of equations is solved stably with the application of a principal component analysis (PCA) approximation. A pseudo inverse of the system matrix is computed using only singular vectors that exhibit non-noise features, i.e. spatial singular vectors containing over 10% energy in the lower 15% of the nominal resolution. Precomputation of this compact inverse model allows for efficient and robust reconstruction. Note that in the cases of 2D phantoms, the inverse model excludes even harmonic information that will negligibly contribute to in-plane reconstructions.

As with most forms of regularization for image reconstruction there is an inherent trade-off between stability, SNR, and resolution/model accuracy. The form of PCA employed in this work is directly analogous to Tikhonov regularization and assuming fewer basis functions is equivalent to increased levels of Tikhonov regularization. In either case, over-regularization will lead to higher SNR but increased blurring and degraded resolution/model accuracy. The thresholds assumed in this work were chosen empirically to provide a good trade-off between added noise and spatial resolution.

For the acquisition scheme of this device, SPIONs located at the *z* = 0 plane do not produce even harmonics. Therefore, for objects which do not extend beyond this central plane, only the center *z*-slice of the forward model and only odd harmonics are used. Thus, for 2D objects (e.g. the point sources in figure 3 and the phantom fMPI time-series images of figure 4), the reconstruction parameters are set to use the odd harmonic frequencies [3, 5, 7, 9]*f*_0_ and the thresholds described above resulted in 198 singular vectors. The model only uses the center *z*-slice of the forward model as the object does not extend beyond this region. For 3D objects (e.g. rodent volume and time-series images), for which signal can come from SPIONs outside the *z* = 0 plane, the reconstruction algorithm utilizes the 2*f*_0_ and 3*f*_0_ harmonic frequencies, 5 slices in *z*, and 201 singular vectors.

The in-plane native spatial resolution of the system with the Synomag-D SPIONs is 3 mm (Mattingly *et al* 2022). Due to the regularization trade-off, the images reconstructed with the forward model PCA method have slightly improved spatial resolution. For the 5-point source 2D phantom in figure 4, this was measured to be 2.55 mm by matching the width of a small phantom’s image to its ground-truth representation convolved with a Gaussian kernel (Mattingly *et al* 2022).

For the phantom images in figure 3, the SNR is calculated as the maximum value in the central 10 × 10 pixel region of the image, divided by the noise determined from the standard deviation in the same region in an empty bore image. A Rician correction scaling factor of $\sqrt{2{\pi }^{2}/3}$ is applied to account for the non-zero mean of the magnitude images (Gudbjartsson and Patz 1995).

### 2.5. Time-series data analysis

The mechanically rotating FFL scanner produces an image every half-rotation. Gain and alignment differences between images acquired in the first half of the rotation (0°–180°, referred to as ‘upper quadrant’ images, i.e. images 0, 2, 4...) versus the second half of the rotation (180°–360°, ‘lower quadrant’ images) necessitated preprocessing with a band-stop filter to remove resulting image time-series artifacts from these differences as well as effects from the half-rotation pause time between sets of 5 continuously acquired images. Representing ‘upper quadrant images’ as ‘1’ and the ‘lower quadrant images’ numbered as ‘0’ with the pause as a blank space, the image time-series takes on a pattern of [10101 10101 ...], which has Fourier contributions at 0.2 and 0.4 cycles per sample, i.e. once every 5 images and half that (second harmonic). The source of the gain and alignment differences was not determined, but is likely due to some unintended parasitic coupling to the rotating magnetic fields (shift and FFL). While elimination of this systematic time-series artifact via hardware refinement would be preferred, we removed these artifacts using a digital band-stop filter at these two frequencies with bandwidth of 0.075 cycles per sample.

**Figure 2.**
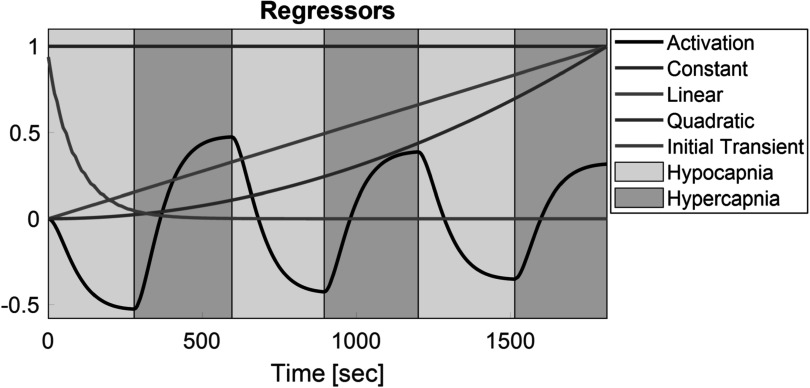
Generalized linear model (GLM) regressor terms: activation, constant, linear, quadratic, and initial transient terms. The activation term models the net functional CBV response including the hypercapnia cycle, CO_2_ response, and SPION decay. For the activation regressor shown here, *τ*_1_ = 51 s and *τ*_2_ = 3100 s.

After this pre-processing filtering step, we fit the time-series using a generalized linear model (GLM) designed to describe the hyper-/hypocapnia induced changes and account for slow temporal drift in the image intensity. The GLM used the regressors shown in figure 2 which included: (1) a constant signal baseline term to allow for spatial variation of each voxel’s baseline blood volume signal, (2) linear and (3) quadratic trends to account for system gain drifts, and (4) an initial transient decay to capture transient warm-up effects at the start of the protocol (${e}^{-t/{\tau }_{d}}$, ${\tau }_{d}\approx 100\,{\mathrm{s}}$, empirically set to 1/3 of the first half period of activation). The final regressor term (5) models the CBV changes expected from the functional challenge. This consisted of a block waveform with the period of the applied hyper-/hypocapnia cycle convolved with a CO_2_ response function (CO_2_RF $=\,{{te}}^{-t/{\tau }_{1}}$) and multiplied by a particle decay term accounting for the blood SPION clearance (${e}^{-t/{\tau }_{2}}$). The CO_2_ response function includes delays along the gas lines, inflation of the lungs, absorption of the gas, delivery from lungs to the brain, and finally, the hemodynamic response of the brain. An optimization was run to find the *τ*_1_ and *τ*_2_ and regressor amplitudes that provide the best fit to the data. The CNR is defined as the peak-to-peak amplitude of the CO_2_RF convolved block waveform (e.g. the amplitude of the 5th regressor term) divided by the standard deviation of the residual error from the GLM.

The activation block design was manually recorded during the experiment with a digital switch whose state was recorded with the imaging data (time-stamped to the data). However, this manual synchronization introduces possible temporal misalignment between the data and the capnic state in addition to any inherent delays between initiating the change in ventilator settings and the gas mixture experienced by the rat. To account for this, a variable delay time Δ*t* was included in the GLM allowing the model fit to shift the activation block function in time. This delay term is coarsely optimized in steps of 5 s, over a range −20 to +20 s to improve the GLM fit.

To analyze the BOLD fMRI time-series, the same GLM analysis is applied but with two changes: (1) the pre-processing band-stop filter step is omitted, as this correction is specific to the MPI imager hardware, and (2) the particle decay term (${e}^{-t/{\tau }_{2}}$) is set to 1, as no tracer is utilized in a BOLD sequence and thus particle washout is not relevant.

For the phantom time-series analysis, the same processing and GLM are used as in the rodent fMPI data, but with the particle decay term and the CO_2_ response function both set to 1, as neither apply. Additionally, an additional linear drift regressor term, which is set to zero when the phantom is out, is included to account for signal-dependent linear drift.

## 3. Results

### 3.1. Image and image time-series sensitivity

**Figure 3.**
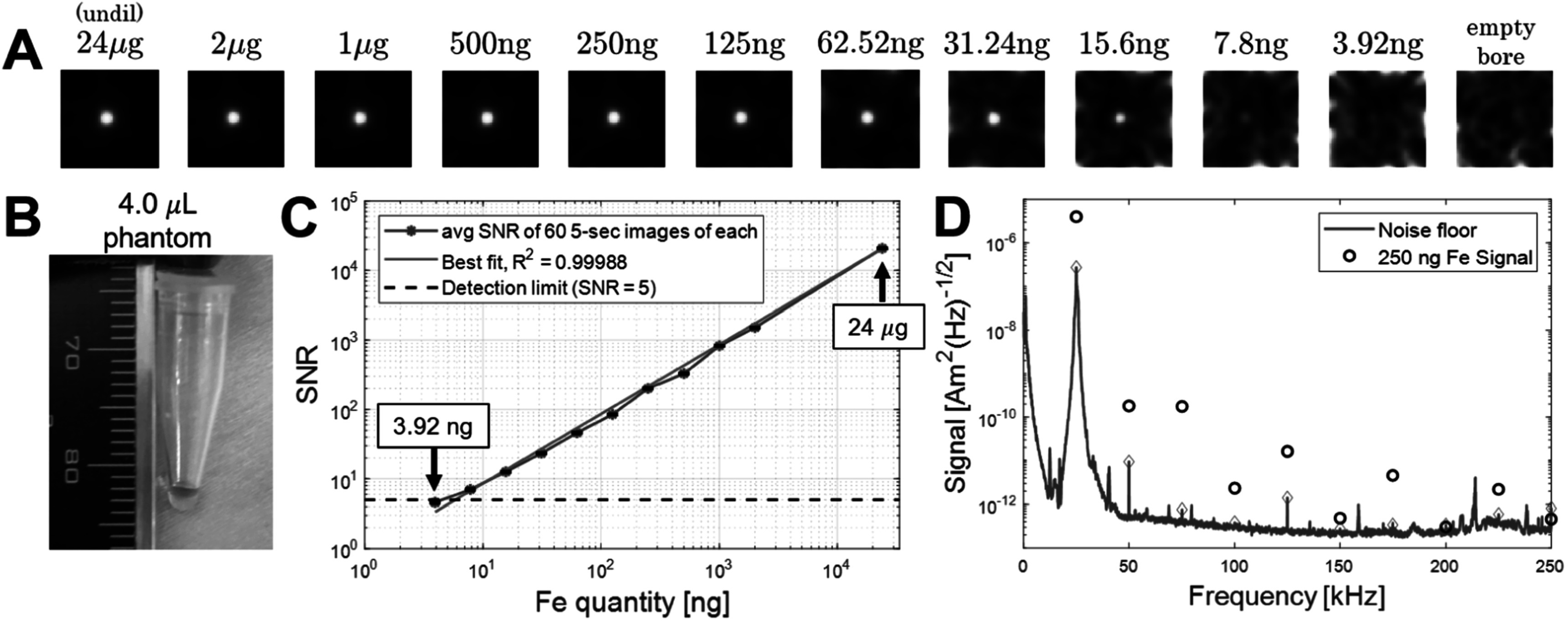
Imager sensitivity and noise characterization. (A) Images of phantoms of 4.0 *μ*l Synomag-D 70 nm (Lot: 16321104-02) SPIONs in a microcentrifuge tube, ranging in Fe quantity from 24.0 *μ*g down to 3.92 ng, 5 s imaging time. These 2D images were reconstructed using a hybrid simulated/measured forward model with parameters as described in the image reconstruction section of methods. Images were smoothed with a 3 mm Gaussian kernel. (B) Photo of one of the 4.0 *μ*l SPION phantoms. (C) Plot of SNR as a function of iron quantity to assess signal linearity and slope (*R* ^2^ = 0.999, slope = 0.86 SNR ng^−1^). (D) Empty-bore noise measurement (standard deviation of the power spectral density, with the 25 kHz drive current on) as a function of frequency (blue trace). Signal levels at the detected harmonics are shown with diamonds. For comparison, open circles show the expected signal levels of a 250 ng sample approximating the expected Fe load in a 3 mm cubic voxel in the resting brain.

Figure 3 shows the minimum detectable tracer quantity visible in a single 5 s image based on a phantom dilution series. The phantoms are 4.0 *μ*l volumes of Synomag-D 70 nm (Micromod, Germany) at the apex of a microcentrifuge tube. Sample dilutions ranged from 24 *μ*g Fe (undiluted tracer) to 3.92 ng, decreasing first to 2.0 *μ*g, and then by factors of 2. The reported SNR is the single 5 s image’s mean SNR determined from 60 measurements of each phantom. A Rician to Gaussian correction (Gudbjartsson and Patz 1995) is applied to account for the non-zero mean of the magnitude images. The signal was linear with concentration as seen by the linear fit with *R* ^2^ = 0.99988 and slope of 0.86 SNR ng^−1^. The sensitivity limit, defined as the concentration at SNR = 5 in the 5 s of imaging data, was reached at an Fe quantity of 5.8 ng. Figure 3D (blue trace) also shows a major source of noise in the measurement: the presence of unstable signal from an empty bore at the frequencies where the SPION’s signal is expected (harmonics of the 25 kHz drive frequency). These spectral peaks disappear when the drive amplifier is turned off revealing a lower thermal noise baseline.

**Figure 4.**
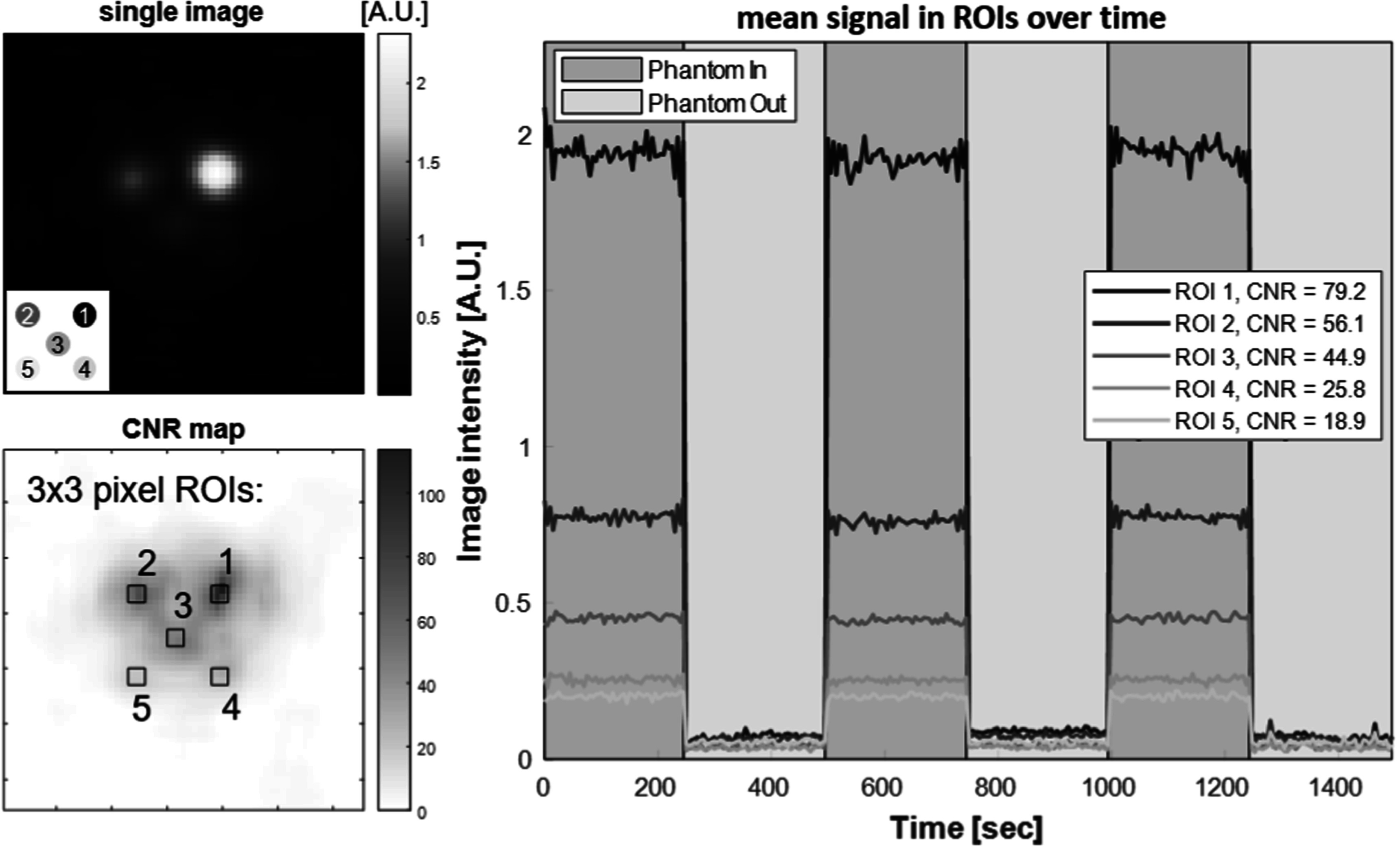
Phantom ‘fMPI’ time-series. A phantom of 5 point-sources with differing SPION concentrations was constructed by pipetting 4.0 *μ*l of SPION solution (Synomag-D 70 nm (Lot:16321104-02) into 3D printed wells. The 0.5× dilution series provided concentrations of (1) 62.5 *μ*g ml^−1^, (2) 31.3* μ*g ml^−1^, (3) 15.6 *μ*g ml^−1^, (4) 7.81 *μ*g ml^−1^ and (5) 3.91 *μ*g ml^−1^ corresponding to Fe masses of 250 ng, 125 ng, 62.5 ng, 31.25 ng and 15.6 ng. The phantom is moved into and out of the imaging plane every 5 min, and images are acquired every 5 s in sets of 5 with an 83% duty cycle. The Fe content of well #3 was designed to mimic the expected Fe change associated with 25% CBV modulation by hypercapnia.

Figure 4 shows the ability of the system to detect temporal changes in an image time-series. A block-design ‘stimulus’ with the same 5 min timing as the hypercapnia study was achieved by moving a phantom in and out of the bore every 5 min during continuous 5 s imaging. The phantom was a 2D array of five wells (4.0 *μ*l each) which varied in dilution to represent a range of relevant physiological voxel Fe mass expected for the rodent CBV experiments. Iron dilution concentrations ranged from 62.5 down to 3.91 *μ*g ml^−1^ (Fe mass per ml water) in 2-fold decrements. The first well had an iron content of 250 ng, approximately the expected Fe load in a 3 mm cubic voxel in the resting brain. Resting brain Fe mass in a 3 mm voxel was estimated to be 210 ng when injected with a 10 mg kg^−1^ Fe dose, assuming 5% gray matter blood volume and 64 ml blood per kg body weight. Thus a 25% CBV change approximately corresponds to the Fe voxel content change from removal of well #3. This is detected with a CNR of 45 in the phantom time-series experiment.

The ‘in’ vs ‘out’ signal difference of the smallest concentration phantom (3.91 *μ*g ml^−1^) is clearly visible in the time-series. Also note that the standard deviation (SD) of the noise in the time-series grows in proportion to the signal level; it is highest for the high concentration phantom (highest signal level). This suggests a significant variance contribution from instrumental gain fluctuations. Fitting the relationship between signal level (S) and time-series standard deviation (*σ*_tot_) to the relationship: ${\sigma }_{\mathrm{tot}}=\sqrt{{\sigma }_{0}^{2}+{\left(\lambda {\mathrm{S}}\right)}^{2}}$, with image noise SD (*σ*_0_) measured to be *σ*_0_ = 0.0133 A.U., provided *λ* = 0.017 with an *R* ^2^ = 0.792. Within the limitations of this 5 data-point fit, this suggests that the thermal variance and signal level-dependent variance sources contribute equally to the time-series for image SNR = 59, which corresponds to a 68 ng Fe source, about a third the Fe quantity expected in a 3 mm cubic resting brain voxel.

### 3.2. Rodent volume imaging

**Figure 5.**
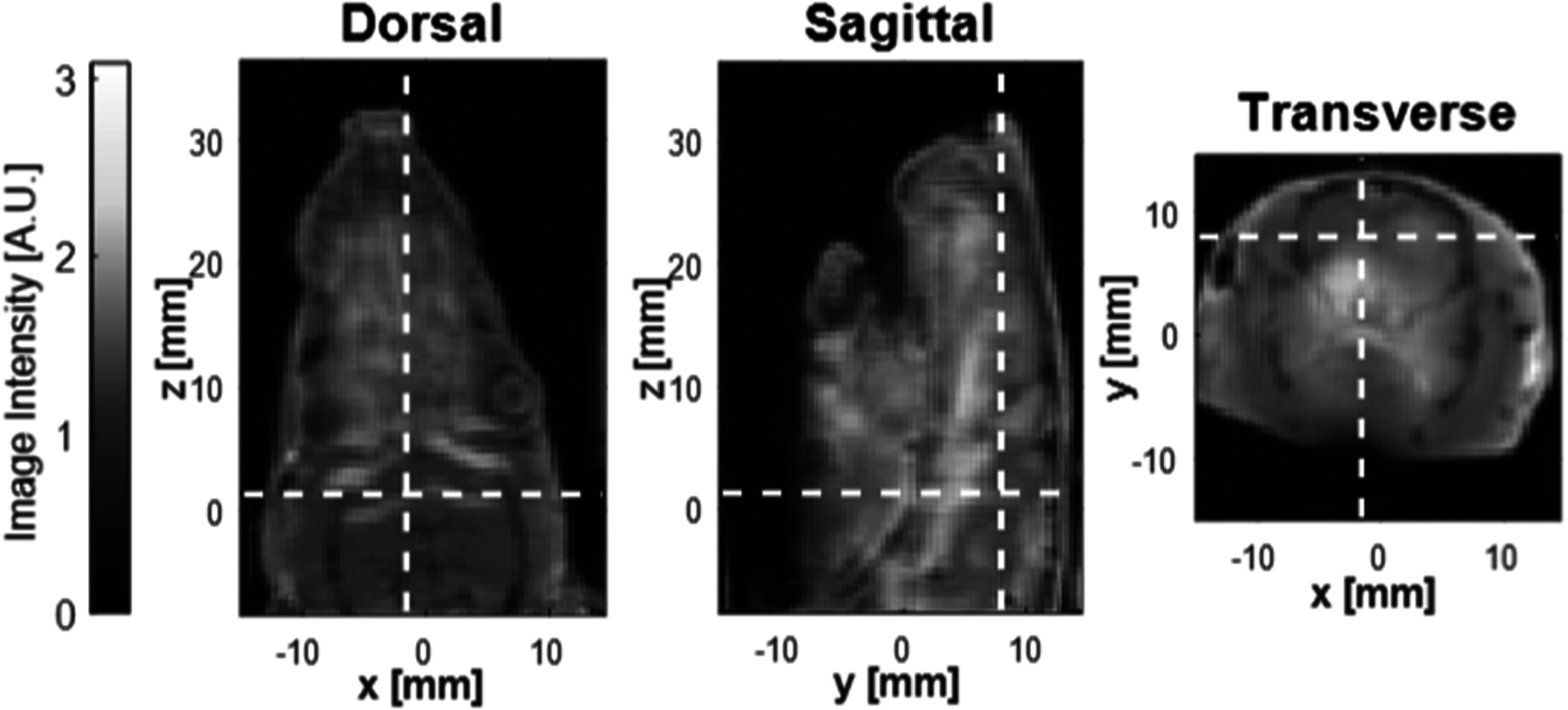
MPI volume scan of rodent. 5 s images acquired at 72 locations of the rat bed along the bore and stacked to form a volume. The MPI images are overlaid on a volume gradient echo MRI scan aligned to the MPI volume using MRI visible fiducials on the MPI rodent bed, which was transported with the animal to the MRI scanner. The white dashed lines indicate the positions of the other two anatomic planes shown.

Figure 5 shows a volumetric image of one rodent formed by concatenating the 5 s images from 72 bed locations, achieved by advancing the motorized bed by 0.5–0.7 mm increments. The MPI volume formed from this multi-slice data set is overlaid on a high resolution anatomical gradient echo volume image taken on a 3T MRI scanner after sacrifice of the animal without moving the animal off the MPI bed/holder. The MRI volume images were aligned to the MPI coordinate system using MR visible fiducials embedded in the MPI bed. The anatomical MPI CBV image is dominated by the large vessels in the rodent head.

### 3.3. Rodent hyper-/hypocapnia experiments

**Figure 6.**
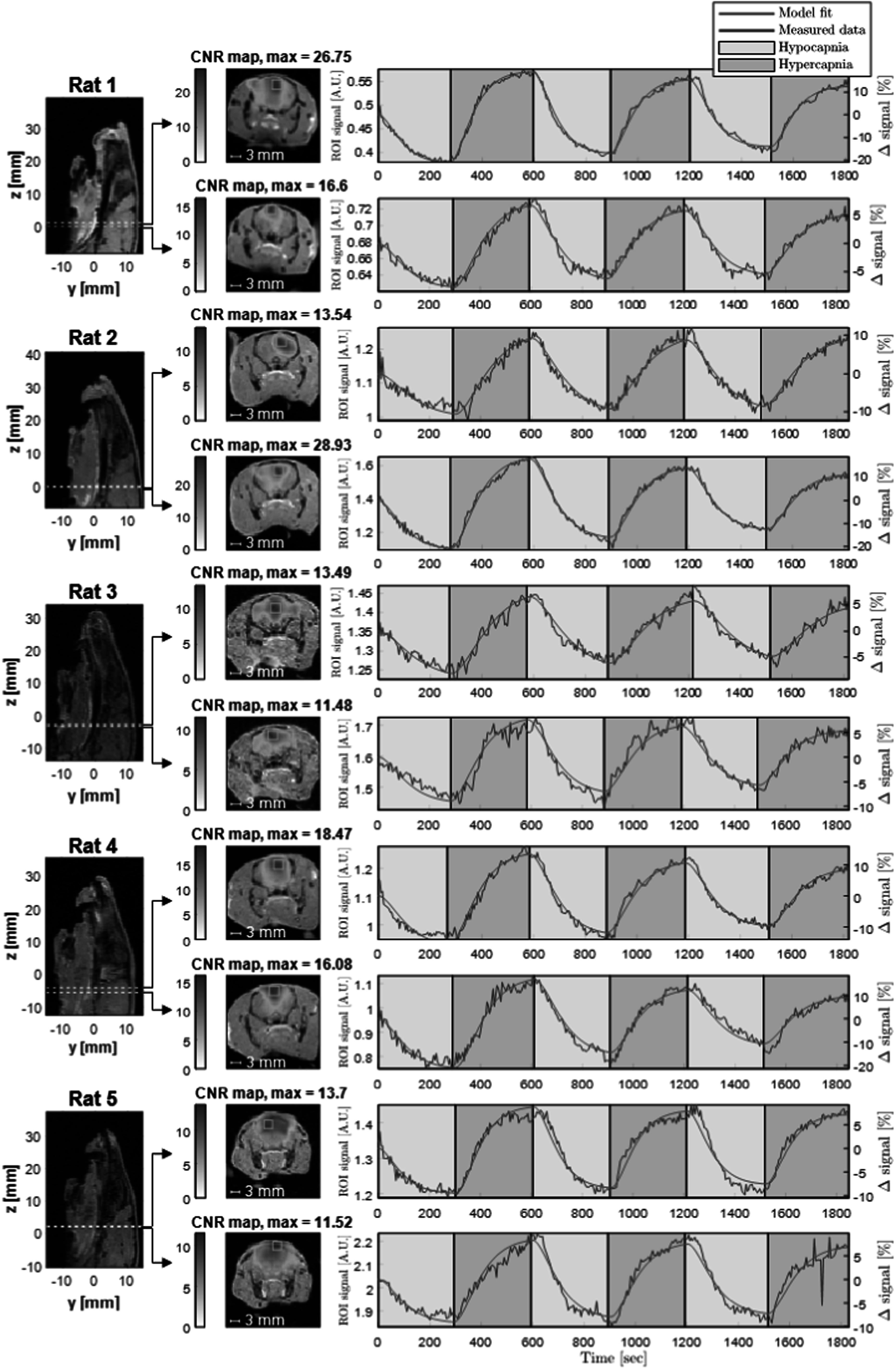
Results from the 10 CBV fMPI experiments in the 5 rats, fitted with a 5 parameter GLM. Each experiment consists of 3 cycles of alternating hypocapnia (yellow) and hypercapnia (orange). (Left) Sagittal slices of the coregistered MRI with dashed lines indicating the positions of the fMPI imaging planes. (Center) CNR maps of the MPI time-series coregistered and overlaid on the transverse plane MRI, with transparency weighted by CNR value. The CNR was defined as the peak-to-peak amplitude of the ΔCBV activation regressor divided by the SD of the residual of the GLM model fit. Maximum-CNR voxel indicated with a blue box. (Right) Time-series of the max-CNR voxel with drift and transient regressor terms subtracted off. Measured data = blue, model fit = red. These data are aquired with 5 s temporal resolution and 2.55 mm reconstructed resolution, and then smoothed with a 3 mm FWHM Gaussian kernel. Percent signal changes ranged from ∼15% to 40% and peak CNR for the 10 experiments was [26.75, 16.60, 13.54, 28.93, 13.49, 11.48, 18.47, 16.08, 13.70, 11.52].

Five Sprague-Dawley rats underwent the full hypercapnic manipulation protocol during MPI imaging. The protocol included two hyper-/hypocapnia experiments per rat, for a total of 10 experiments. Each experiment consisted of an injection and three 10 min cycles of alternating hypo- and hypercapnia. Figure 6 shows the resulting map of the ΔCBV activation amplitude based on each voxel’s fit to the 5 regressor GLM. Within the brain, the percent signal changes in each experiment were [39.58, 14.53, 19.80, 38.68, 14.85, 16.52, 28.87, 37.92, 18.26, 17.32]% with an average value of 24.63%. The activation map was thresholded at a Bonferroni corrected p-value of <10^−12^. The voxel with the largest activation contrast-to-noise ratio, (CNR, defined as the ratio of peak-peak activation regressor amplitude to the SD of model residual) is shown on the map along with the signal time-course for that voxel. The maximum positive CNR in each experiment was [26.75, 16.60, 13.54, 28.93, 13.49, 11.48, 18.47, 16.08, 13.70, 11.52] (average 17.06). The GLM fit determined a measured blood SPION decay time constant, *τ*_2_, corresponding to a blood half-life: ${t}_{1/2}={\tau }_{2}{ln}(2)$. The average measured *t*_1/2_ was 48 min, consistent with prior findings for Synomag-D (Szwargulski *et al* 2020, Liu *et al* 2021).

**Figure 7.**
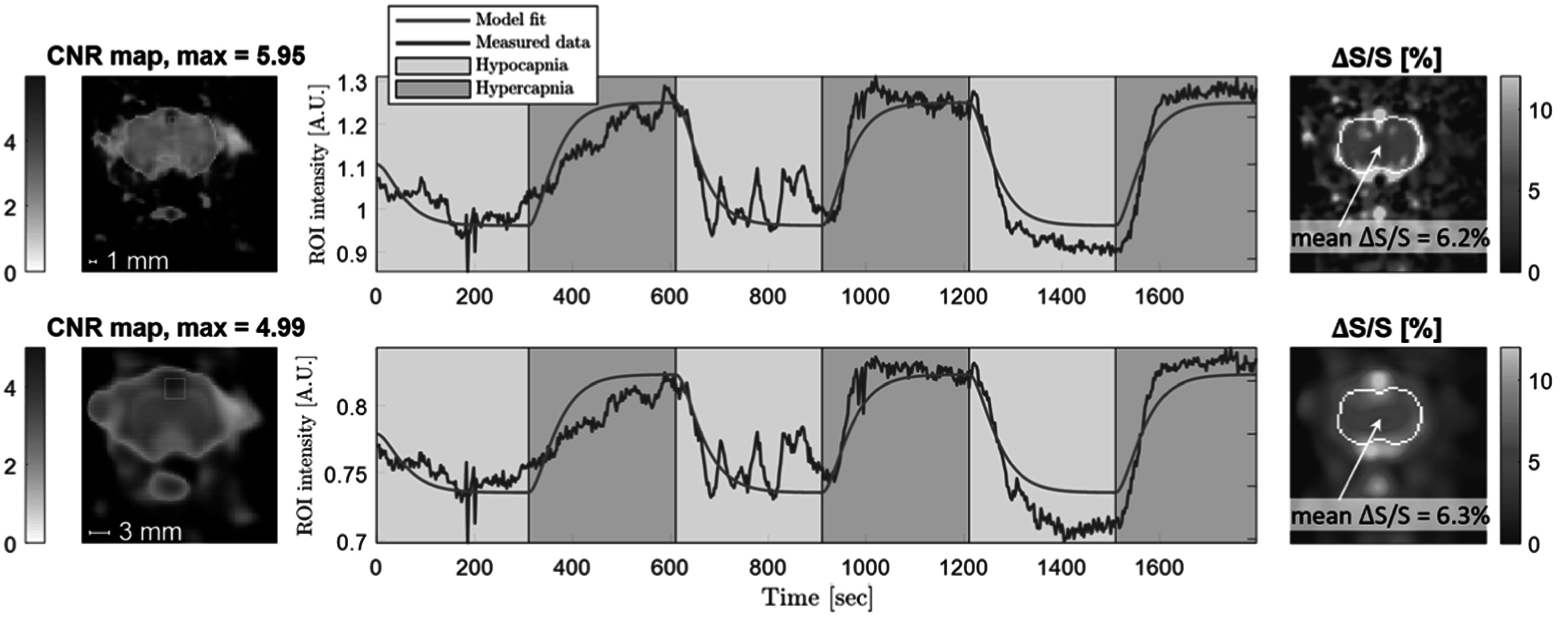
Results from BOLD fMRI at 9.4T of a single rat using the same hyper-/hypocapnia protocol without the injection of SPIONs. The 0.375 mm nominal resolution gradient echo EPI data was spatially smoothed using either a 1 mm (first row) or 3 mm (second row) FWHM Gaussian kernel. Scale bars in each image show the kernel size. The CNR map is overlaid onto the first image in the time-series. The time-series is shown for the maximum CNR voxel as well as its fit to the GLM. This GLM did not include a SPION decay term (${e}^{-t/{\tau }_{2}}$). The right column shows maps of Δ*S*/*S*, with a white outline indicating a segmentation of the parenchyma for assessing Δ*S*/*S* without BOLD signal venous artifacts.

Figure 7 shows data from an additional animal studied with BOLD fMRI using a 9.4T scanner with the identical hyper-/hypocapnia protocol. The fMRI time-series was smoothed from its native 0.375 mm nominal resolution to 1mm and 3 mm prior to fitting with the same GLM but lacking the SPION decay term. The brain voxels with peak CNR were marked and plotted (peak CNR = 5.95 and 4.99 for 1 mm and 3 mm smoothing, respectively).

## 4. Discussion

We induced alternating periods of hypo- and hypercapnia, an established method for modulating brain vasodilation and blood flow (Kety and Schmidt 1948, Raper *et al* 1971, Ito *et al* 2000), to demonstrate the ability of MPI time-series to monitor the cerebral hemodynamic changes associated with brain function. The static signal levels and the signal changes observed *in vivo* were roughly consistent with measurements in phantoms with SPION concentrations matching the expected resting blood levels and their changes with hypercapnia.

For example, the injected Fe content in a resting 3 mm voxel is expected to be 210 ng. The static phantom with 250 ng Fe from figure 3 showed a signal level of 2.2 A.U. and SNR = 200. After the first injection, the first image from the time-series had a parenchymal brain signal of 1.54 A.U. and SNR of 99.9, averaged across the 5 rats. The second injection yielded a signal and SNR of 2.07 A.U. and 134. For the dynamic phantom experiment, the CNR of the 250 ng source was only 80 (compared to SNR = 200 in the static image). This suggests a signal level-dependent instrumental instability. Eliminating this 2x sensitivity reduction is an obvious target for future instrument development.

The expected voxel Fe content change induced by the inhalation protocol is 52.5 ng, most closely matching our 62.5 ng phantom. The dynamic phantom experiment detected the periodic removal of this amount of iron with a CNR = 44.9, which was higher than the rodent hypercapnia experiments (CNR_avg_ = 17 with individual runs ranging from 11.48 to 28.93). This difference between the dynamic phantom and *in vivo* measurements is likely due to an additional *in vivo* noise source, physiological noise: time-series variance induced by random-appearing physiological modulations of the signal.

The range of *in vivo* CNR in the rodent hypercapnia experiments (11.48–28.93) and relatively high animal-to-animal variance observed could partially arise from differences in animal placement within the imaging FOV as well as the position along *z*. The positions along *z* were selected based on educated guesses regarding the intended location within the brain, as the MRI co-registration was not available until after animal sacrifice. This led to variation in the location of the slice. The animal bed and breathing apparatus necessitated positioning of the brain near the top edge of the FOV. The receive coil and FOV (determined by the shift current amplitude) was just large enough for the animals studied. Image reconstruction and analysis would be simplified with a larger FOV. As rat #5 sat particularly high in the FOV, a 12% increase in FOV (33.6 cm) was attempted by pushing to the limits of our shift amplifier’s voltage range. Visual improvement is observed in the reconstruction as seen in the homogeneity of the CNR map over the whole brain. Further, some variation might have arisen from batch differences in the detection properties of the SPIONs. Animal 1 used batch #1 of the particles, and animals 2 through 5 used batch #2, which showed lower MPI detection sensitivity in phantom measures. Sources of run-to-run variability (differences between the two experiments for a single rat) are suggested from the observation that the first and second experiments differed in CNR by an average of 33%. One important difference between experiments is the second SPION dose and variable time between injection and start of the time-series data collection, such that the baseline SPION blood concentration differs between the two experiments. The CNR and Δ*S*/*S* tended to be correlated for the experiments, suggesting that some of the run-to-run variance might be due to variations in the hypo-/hypercapnic state. The high run-to-run variance is not seen in the phantom imaging tests. Another potential cause is reconstruction artifacts arising from high blood volume regions out of plane, which could be subject to small variations in *z* position; these artifacts are not an intrinsic limitation and can be resolved in future development. Finally, the rats’ vitals fluctuated under anesthesia over the course of the multi-hour protocol, which could also contribute to the differences.

The CNR of a block design activation study is defined as the ratio of the Δ*S* between the activated and baseline states to the SD of the time-series noise (*σ*_tot_): CNR = Δ*S*/*σ*_tot_. Our results showed that the hypercapnia protocol modulated the 3 mm voxel MPI signal by an average Δ*S*/*S* = 25%. The observed percent signal change is consistent with rodent CBV changes measured with MRI under similar hypercapnia protocols. For example, after 5% CO_2_ administration, Lu *et al* (2009) saw a ∼35% change in the rat's CBV (Lu *et al* 2009). For the BOLD-fMRI experiment, the average parenchymal voxel Δ*S*/*S* = 6%. The difference between Δ*S*/*S* observed in fMPI and in BOLD-fMRI is expected, due to the differing detection mechanisms. fMPI directly measures the ΔCBV as reported by the change in SPION content, while the BOLD-fMRI mechanism is an indirect measurement of blood oxygenation which is susceptible to background signal modulations. The mean Δ*S*/*S* in the parenchyma is reported to exclude BOLD signal venous artifact effects.

The CNR can be rewritten in terms of the time-series SNR (tSNR = *S*/*σ*_tot_): CNR = (Δ*S*/*S*) × tSNR. Since we performed only a single study with 9.4T MRI, we rely on the considerable literature on rodent fMRI to provide a typical CNR for a hypercapnia challenge detected with MRI. Seehafer *et al* (2010) report a tSNR in the rat brain during fMRI at 7T and 11.7T of 43 and 57 respectively for 0.4 mm in-plane resolution (and 2 mm thick slice). With this tSNR, a typical BOLD fMRI task-induced change of Δ*S*/*S* = 2% would be seen with a voxel CNR of near unity. Hypercapnia generates a larger BOLD effect Δ*S*/*S* than task induced fMRI. Brevard *et al* report Δ*S*/*S* = 3.2% in a whole-cortex ROI detected in the anesthetized rat at 4.7T fMRI during hypercapnia with 5% CO_2_ and a whole-cortex CNR of 7.3 (Brevard *et al* 2003). This combination of Δ*S*/*S* and CNR corresponds to a tSNR of 228 for the whole-cortex ROI. The lower voxel tSNRs reported by Seehafer for the higher resolution imaging would provide CNR = 1.4 and 1.8 for Δ*S*/*S* = 3.2%. Our single BOLD fMRI experiment showed a voxel CNR maximum of 5 performed at 9.4T after smoothing to the spatial resolution to 3 mm.

SPION injections are commonly used in fMRI to generate CBV contrast fMRI. In this case, the signal change during neuronal activation is negative (the signal goes down during activation or hypercapnia). This is opposite to the more frequently used BOLD mechanism (signal goes up during neuronal activation or hypercapnia). Therefore, there will be some field strengths and/or SPION doses where the SPION CBV effect cancels with the BOLD effect. We did not pursue SPION-based CBV in our 9.4T fMRI for this reason.

Our current fMPI instrument does not have a spatial resolution comparable to or as flexible as that of high-field rodent MRI. This will limit the impact of fMPI in rodents, at least pending improvement in spatial resolution. However, the primary goal in demonstrating rodent fMPI is not to compete with rodent fMRI, but rather as a critical step in the pathway to assessing fMPI as a potential clinical tool. Human fMRI studies are commonly acquired at 3 mm (Huettel *et al* 2014), and are regularly smoothed, in the range of 4–12 mm (Sacchet and Knutson 2013, Triana *et al* 2020). Human-scale MPI has been predicted and demonstrated with 5–7 mm resolution (Mason *et al* 2017, Graeser *et al* 2019) and offers many avenues for future development. Efforts to improve MPI resolution focus on the two major determinants of MPI spatial resolution: the gradient strength of the FFL and the transition width of the particle's magnetization curve. Research (Yu *et al* 2017) and commercial (Magnetic Insight Inc.) product instruments have been reported with 2-fold higher FFL gradients (6.3 and 5.7 T m^−1^, respectively) than ours, and so even at the rodent scale, our system does not demonstrate the ceiling of MPI’s capability. Magnetic nanoparticle structures with up to 10-fold higher native resolution are also under development (Tay *et al* 2021), offering further improvement of both preclinical and clinical-scale MPI spatial resolution.

The differing spatial resolution also complicates the comparison of CNR in the two modalities. The 9.4T was selected as a typical high-end rodent fMRI acquisition, and used standard TR, TE and image resolution. While we could have acquired the fMRI with a lower field scanner with resolution closer to 3 mm, using lower field strengths would only reduce the CNR of the measurement (Triantafyllou *et al* 2006). We attempted to make the CNR comparison by smoothing the fMRI data to that of the fMPI. For uncorrelated noise, smoothing should improve tSNR and thus CNR by a factor of $\sqrt{V}$, where *V* is the ratio of the post- to pre-smoothing voxel volume. However, the tSNR gains in practice are limited by the spatial correlation of the physiological noise (Triantafyllou *et al* 2006).

The 5 s temporal resolution chosen for this instrument is not an inherent limitation of MPI. It is largely constrained by our choice to use a mechanically rotating FFL, which was chosen to facilitate scaling the device to a human scale, for which our rodent device is a small-scale prototype. Additionally, we note that the 5 s temporal resolution seems poor, but in fact, Nyquist samples the hemodynamic response (about 10 s in length) and the 0.01–0.1 Hz band of resting state hemodynamic fluctuations. If higher sampling rates are needed, either for other applications or to capture faster than typical hemodynamics, the gantry speed of our imager could be increased up to 2×. Beyond that, a non-mechanical shifting/rotation is likely required.

The CBV contrast in MPI, like many tracer methods, is a positive contrast, whereas in fMRI, CBV studies with SPIONs provide a negative contrast. Thus, voxels with a high fraction of blood (such as those containing the Circle of Willis) appear very bright in MPI but are dephased (dark) in the fMRI time-series. These bright features are seen in the volume MPI image in regions of large vessels (which have 20-fold the CBV of parenchyma). The hypercapnic CBV changes in these large vessel regions are smaller and were observed to have the opposite sign of the parenchymal changes, allowing their removal from the functional maps. Nonetheless, the poor spatial resolution and out-of-plane contamination not sufficiently addressed in the image reconstruction might modulate the detected CBV response in regions adjacent to large vessels. This could contribute to the variation across the brain and across animals seen in the fMPI activation maps compared to the fMRI maps. This could be addressed with an improved detection system, for example from more temporal harmonics and the addition of a transverse reception coil.

Of note, the responses to the change in hypercapnic state do not quite reach a steady state in the 5 min blocks. However, the 5 min blocks were sufficient to see and model the response, and longer blocks would only improve the CNR. While the hemodynamic response is much faster, the observed slower responses are expected: what is being measured is the total CO_2_ response, of which the hemodynamic response is only a small part (and includes delays along the gas lines, lung inflation and absorption, and delivery to the brain). In neuronal activation studies, only the hemodynamic response function (HRF) is relevant and therefore allows for much faster block design studies (typically 30 s blocks are used in fMRI).

The fMPI results presented do not benefit from the many decades of technical polishing found on modern fMRI scanners. For example, the variance in our phantom time-series is dominated by unstable harmonics originating in our drive amplifier system rather than thermal noise. Eliminating this with either improved filtering or use of a more linear amplifier would increase our tSNR by a factor of about 2. Without that source of variance, the system is limited by a combination of thermal noise from the preamplifier and the copper losses in the receive coil’s windings, both of which might be improved. This contrasts with MRI where these sources are below that of the noise from resistive losses in the body. Finally, since the MPI signal is detected through Faraday induction, sensitivity might be improved by optimizing the drive frequency and amplitude (Tay *et al* 2020), or anatomically optimized receive coils (Graeser *et al* 2020).

## 5. Conclusions

The rodent hypercapnia experiments suggest that functional MPI can detect the temporal hemodynamic changes expected to accompany brain activation with a high contrast-to-noise ratio for studies where high spatial resolution is not critical. The observed average CNR of the CBV time-series was roughly 2–6 fold higher than that observed in 9.4T fMRI with an additional factor of 2 potentially available from easily identified instrumental improvements.

In conclusion, we introduced a new modality for functional brain imaging based on detection of CBV using magnetic particle imaging of blood-pool iron oxide nanoparticles. While the limited spatial resolution is problematic for preclinical studies, demonstration of rodent fMPI time-series imaging is an important stepping stone to assess fMPI as a potential clinical tool, where 3 mm resolution is comparable to many human fMRI studies. Our approach leverages the high magnetic moment of the SPION agents and the direct detection of the blood pool without the dilution of signal from other sources. We show that this approach could allow for higher sensitivity to functional hemodynamic changes than fMRI methods for studies where sensitivity is valued over spatial resolution. We report the first fMPI time-series for imaging hemodynamic modulation and characterize the *in vivo* signal and noise levels compared to phantom experiments and those seen in rodent fMRI experiments. The presented *in vivo* rodent brain image time-series demonstrate the potential of MPI as a functional imaging modality, which could expand the range of methods available for neuroscience and clinical studies.

## Data Availability

The data that support the findings of this study are openly available at the following URL/DOI: %%%https://github.com/ericamason/Rat-fMPI.

## Ethical statement

These experiments were carried out under approved protocol #2016N000016 from Massachusetts General Hospital’s Institutional Animal Care and Use Committee (IACUC), and adhered to ARRIVE guidelines.

## Author contributions

**Erica Mason:** Conceptualization, Methodology, Software, Formal Analysis, Investigation, Resources, Data Curation, Writing—Original Draft, Visualization. **Eli Mattingly:** Conceptualization, Methodology, Software, Formal Analysis, Investigation, Resources, Data Curation, Writing—Review and Editing, Visualization. **Konstantin Herb:** Conceptualization, Methodology, Software, Investigation, Resources, Data Curation. **Stephen Cauley:** Software, Formal Analysis, Writing—Review and Editing. **Monika Śliwiak:** Investigation, Resources. **John Drago:** Investigation, Resources. **Matthias Graeser:** Resources, Writing—Review and Editing. **Emiri Mandeville:** Investigation, Resources. **Joseph Mandeville:** Conceptualization, Methodology, Investigation, Resources, Data Curation, Writing—Review and Editing, Supervision, Project Administration, Funding Acquisition. **Lawrence Wald:** Conceptualization, Methodology, Writing—Original Draft, Supervision, Project Administration, Funding Acquisition.

## Competing interests

EEM is currently an employee of Magnetic Insight, Inc. LLW and SFC receive research funding from Siemens Healthineers. LLW is a co-founder, equity holder and consultant for Neuro42 Inc., a company pursuing point of care brain MRI.

## Appendix. Supplementary data and code

Image time-series data at each processing step for all ten fMPI experiments and for the fMRI experiment are available on https://github.com/ericamason/Rat-fMPI as .mat files. The available data include the raw image time-series, (named ‘ImageTimeSeries’), the band-stop filtered time-series (‘ImageTimeSeries_Filt’), and the spatially smoothed time-series (‘ImageTimeSeries_FiltSmooth’) that used Gaussian smoothing kernel size (‘conv_kernel_size_mm’). The corresponding time vector (‘TimeSec0’), activation blocks (‘CapniaTrigSeries’), and optimized values for *τ*_1_ (‘Tau1’), *τ*_2_ (‘Tau2’) and the delayed time (‘Delay Time’) are saved in the .mat file for each.

The code to process the supplied data is also provided at https://github.com/ericamason/Rat-fMPI. This code loads the desired .mat file, solves the general linear model, and plots the CNR map and maximum CNR voxel time-series data. The ‘RunMe.m’ function initiates the full analysis and plotting.
